# Encoding and Retrieval Interference in Sentence Comprehension: Evidence from Agreement

**DOI:** 10.3389/fpsyg.2018.00002

**Published:** 2018-01-19

**Authors:** Sandra Villata, Whitney Tabor, Julie Franck

**Affiliations:** ^1^Psycholinguistics Laboratory, Department of Psychology, Université de Genève, Geneva, Switzerland; ^2^Department of Psychology, University of Connecticut, Storrs, CT, United States; ^3^Haskins Laboratories, New Haven, CT, United States

**Keywords:** similarity-based interference, retrieval, encoding, long-distance dependencies, working memory, agreement, ACT-R, self-organized sentence processing

## Abstract

Long-distance verb-argument dependencies generally require the integration of a fronted argument when the verb is encountered for sentence interpretation. Under a parsing model that handles long-distance dependencies through a cue-based retrieval mechanism, retrieval is hampered when retrieval cues also resonate with non-target elements (retrieval interference). However, similarity-based interference may also stem from interference arising during the encoding of elements in memory (encoding interference), an effect that is not directly accountable for by a cue-based retrieval mechanism. Although encoding and retrieval interference are clearly distinct at the theoretical level, it is difficult to disentangle the two on empirical grounds, since encoding interference may also manifest at the retrieval region. We report two self-paced reading experiments aimed at teasing apart the role of each component in gender and number subject-verb agreement in Italian and English object relative clauses. In Italian, the verb does not agree in gender with the subject, thus providing no cue for retrieval. In English, although present tense verbs agree in number with the subject, past tense verbs do not, allowing us to test the role of number as a retrieval cue within the same language. Results from both experiments converge, showing similarity-based interference at encoding, and some evidence for an effect at retrieval. After having pointed out the non-negligible role of encoding in sentence comprehension, and noting that Lewis and Vasishth’s (2005) ACT-R model of sentence processing, the most fully developed cue-based retrieval approach to sentence processing does not predict encoding effects, we propose an augmentation of this model that predicts these effects. We then also propose a self-organizing sentence processing model (SOSP), which has the advantage of accounting for retrieval and encoding interference with a single mechanism.

## Introduction

One characteristic property of natural language is that it allows for long-distance dependencies: elements that are not adjacent in the input may nonetheless be related to one another. Successful language comprehension thus requires non-adjacent constituents to be accessed for semantic interpretation. Object relative clauses are well-known examples of long-distance dependencies: in these configurations, the internal object of the verb does not occupy its canonical post-verbal position, but it is fronted to the beginning of the clause, as in (1).

(1) The waiter that the dancer surprised drank a rum cocktail.

Upon encountering the verb of the relative clause (*surprised*), a successful understanding of the sentence requires the fronted object to be retrieved and integrated with its verb. Several studies have provided evidence that retrieval is *cue-based* or *content-addressable*, meaning that it is driven by cues that allow the parser to access the intended element based on its content, rather than scanning all the elements in memory in sequence (e.g., McElree and Dosher, 1989; McElree, 2000, 2006; McElree et al., 2003; Martin and McElree, 2008; Van Dyke and McElree, 2011). On the cue-based hypothesis, the retrieval cues are triggered at the verb and form a subset of the target’s features: only features that are cued at the verb constitute retrieval cues (e.g., if the to-be-retrieved element is feminine, but the verb does not carry gender agreement in the syntactic context, feminine will not be a retrieval cue). Content-addressability makes retrieval efficient. However, this efficiency comes at a cost: cue-based retrieval is sensitive to *similarity-based interference* from elements in memory whose featural specification also matches the retrieval cues at the verb (henceforth, *retrieval interference*; e.g., Van Dyke, 2002, 2007; McElree et al., 2003; Van Dyke and Lewis, 2003; Lewis and Vasishth, 2005; McElree, 2006; Van Dyke and McElree, 2006, 2011). The situation in which retrieval cues resonate with multiple items in memory is referred to as *cue-overload* and is considered one of the major causes of retrieval failure (e.g., Watkins and Watkins, 1975; Anderson and Neely, 1996; Nairne, 2002; Öztekin and McElree, 2007).

Research in the memory domain has uncovered another critical source of similarity-based interference, which arises when the target element shares one (or more) features with other elements in memory. This situation is referred to as *encoding interference* (e.g., classical spatial and word list studies: Oberauer and Kliegl, 2006; Oberauer and Lange, 2008; sentence processing studies: Barker et al., 2001; Gordon et al., 2001, 2004; Hofmeister and Vasishth, 2014; Kush et al., 2015) and, unlike retrieval interference, arises regardless of whether the overlapping feature is a retrieval cue. One possible mechanism that has been proposed to account for encoding interference is *feature overwriting*, which assumes feature competition amongst similar elements in memory, such that the element losing the competition results in a degraded memory representation (Nairne, 1990, 2002; Oberauer and Kliegl, 2006). Although retrieval and encoding interference are distinct at the theoretical level, it is difficult to disentangle the two at the empirical level. The difficulty arises because encoding interference arguably also negatively impacts retrieval: by decreasing the distinctiveness and the quality of memory representations, for example, encoding interference could reduce retrieval probability (see also Jäger et al., 2015 for a discussion). The possibility that interference arises at encoding instead of, or in addition to, at retrieval would have important consequences for current prominent models of sentence comprehension that lack a mechanism for generating encoding interference (e.g., ACT-R, Lewis and Vasishth, 2005).

In what follows, we first briefly summarize the empirical evidence for retrieval and encoding interference and the challenges to empirically disentangling the two. We then present two empirical studies on gender and number agreement conducted, respectively, in Italian and English with the aim of teasing them apart. To anticipate the results, we report evidence for similarity-based interference at encoding and retrieval, although the latter was weaker than the former. In the Section “General Discussion,” we discuss two models that can generate both encoding and retrieval interference. The first one is ACT-R (Lewis and Vasishth, 2005) in which retrieval interference is generated by the well-known *fan effect*, responsible to reducing retrieval probabilities for chunks that share the same retrieval cue. We propose that encoding interference can be captured in ACT-R by an additional mechanism we will refer to as *activation leveling*, responsible for equalizing the activation levels of elements sharing a feature. The second model is a self-organized parsing model (SOSP, Tabor and Hutchins, 2004; Smith et al., in press) in which both encoding and retrieval interference follow from general feature-based structure building principles. This model thus has the advantage of capturing both types of interference through the same mechanism.

### Evidence for Retrieval Interference

Traditionally, difficulties manifesting at the region in which retrieval is supposed to be triggered have been interpreted as resulting from *retrieval interference*: when the retrieval cues are not unique to the to-be-retrieved element, the probability of a successful retrieval is lowered, thus increasing retrieval latency at the integration region in on-line measures (because the system must re-retrieve after an error) and decreasing comprehension accuracy in off-line measures (e.g., Anderson et al., 2004; Lewis and Vasishth, 2005; McElree, 2006). For example, using an eye-tracking procedure, Van Dyke (2007) showed that in structures in which the subject and the verb were separated by a relative clause (e.g., *The pilot remembered that the lady who was sitting near the smelly* ***seat/man*** *moaned about a refund*), an element inside a prepositional phrase embedded in the relative clause caused longer regression path times at the region following the critical verb (*moaned*) and lower comprehension accuracy when it was a semantically plausible subject for the verb (*man, animate*) than when it was not (*seat, inanimate*), in virtue of its animacy. Similar evidence was gathered when the target and the distractor were similar in terms of their syntactic roles: a distractor occupying a subject position was found to generate longer reading times at the verb (where the subject needs to be retrieved) and lower comprehension accuracy than a distractor occupying a prepositional object position (Van Dyke and Lewis, 2003; see also Van Dyke and McElree, 2011 for similar findings). Similarity between the subject and the distractor in terms of agreement features were also found to affect agreement processing. In a recent self-paced reading study on subject-verb agreement dependencies in French object relatives, Franck et al. (2015) reported faster reading times at the verb of the object relative when the subject and the object had different numbers as compared to when they had the same number (e.g., *Jérôme speaks to the prisoner-SG/prisoners-PL that the guard-SG takes out-SG sometimes in the yard*; see Adani et al., 2010, 2014 for similar findings in children).

Since the on-line effects found in these studies were attested at the critical retrieval region or right after it, these results were taken as evidence for interference arising at retrieval. However, neither longer reading times at the retrieval region nor lower comprehension accuracy can be taken as conclusive evidence for retrieval interference, since retrieval may also be hampered as a result of *encoding interference*: when the target item shares one (or more) features with other elements in memory, the target and distractor memory traces may interact before the verb arrives, for example, blending their encodings, and this may be the cause of erroneous retrievals and lower comprehension accuracy.

To our knowledge, only two studies actually provide unequivocal evidence for retrieval interference. The first one, conducted by Van Dyke and McElree, 2006, relies on a memory load paradigm combined with a self-paced reading task. The authors manipulated the retrieval cues at the verb such that they either did or did not uniquely identify the target in virtue of the verb’s semantic constraints (Memory load: *table, sink, truck* – Sentence: *It was the boat that the guy who lived by the sea* ***sailed/fixed*** *in two sunny days*). When the retrieval cues were not unique to the target (e.g., *fixed*), they matched all the elements in the memory load. The authors observed longer reading times at the critical verb and lower comprehension accuracy in the cue-overload condition (*fixed*) than in the non-cue-overload condition (*sailed*). Since the memory load was kept constant across conditions, the observed difference can only be attributed to semantic interference at retrieval (this finding does not, of course, allow us to conclude *against* the possible additional role of encoding interference in sentence comprehension in general, as also noted by Jäger et al., 2015). The second data point that unequivocally points to retrieval interference comes from a study on children. In a sentence-picture matching task, Belletti et al. (2012) reported higher comprehension accuracy for object relative clauses in Hebrew speaking-children when the subject and the object had different genders. However, no effect of gender similarity was observed for Italian children. Crucially, while in Hebrew the verb agrees in gender with the subject, therefore providing a subject retrieval cue, in Italian it does not. Since the facilitatory effect of gender mismatch is exclusively attested in Hebrew, when gender is a retrieval cue at the verb, these findings suggest that the gender interference effect arises at retrieval.

Finally, we want to briefly comment on results from Wagers et al. (2009) and much subsequent work, which has failed to observe a match effect of agreement features in grammatical sentences (see Jäger et al., 2017 for a meta-analysis) but found them in ungrammatical sentences – the so-called “grammaticality asymmetry”: participants read the word immediately following the relative verb faster when the verb incorrectly agreed with the object (e.g., *^∗^The musicians who the reviewer praise…*) than when neither the object nor the subject matched the number of the verb (e.g., *^∗^The musician who the reviewer praises…*). Wagers et al. (2009) note that their findings can be accounted for if cue-based retrieval is triggered only when an agreement error is detected. However, this restriction would require cue-based retrieval approaches to find alternative explanations for many grammatical-sentence processing phenomena that they are otherwise in a good position to explain (see, for example, Lewis and Vasishth, 2005; Badecker and Kuminiak, 2007). Moreover, recent evidence supports the position that while there is a grammaticality asymmetry, there is also small-magnitude but reliable competition in the control conditions of the grammatical cases (e.g., N1-Sg N2-Sg V-Sg) which Wagers et al. (2009) failed to detect (Franck et al., 2015; Villata and Franck, 2016; Nicenboim et al., 1234). This suggests that cue-based retrieval is also at work in grammatical sentences. Wagers et al. (2009) point out that if cue-based retrieval is assumed to apply across-the-board, then an additional assumption is needed to explain the grammaticality asymmetry. They note that one such assumption is supra-linear constraint combination (e.g., Gillund and Shiffrin, 1984; Hintzman, 1988; among others). The supra-linear approach makes it so that, when most of the constraints align, as they do in grammatical sentences, the grammatical parse strongly outcompetes any non-grammatical alternatives. This assumption is an arbitrary addition to current cue-based approaches. In the Section “General Discussion,” we argue that self-organized sentence processing (SOSP) offers a principled reason why constraints might be expected to combine supra-linearly.

### Evidence for Encoding Interference

Conclusive evidence for interference that cannot arise at retrieval, and thus must arise at encoding, comes from studies showing effects of similarity between a target and a distractor in terms of features that cannot serve as retrieval cues at the verb. In a series of self-paced reading experiments on relative clauses, Gordon et al. (2001, 2004) reported that the well-attested disadvantage of object relatives as compared to subject relatives was reduced or even eliminated when the subject and the object were of different syntactic kinds (e.g., a pronoun and a definite description or a proper name and a definite description) as compared to when they were of the same syntactic kind. Faster reading times at the verb and higher comprehension accuracy were observed in mismatching conditions (e.g., definite description vs. pronoun, *The barber that you admired climbed the mountain*) as compared to match conditions (e.g., two definite descriptions, *The barber that the lawyer admired climbed the mountain*). Since the distinction between definite description and pronoun is not cued by the verb, the facilitation effect of mismatch cannot lie in the cue-based retrieval process directed at satisfying the constraints of the verb. Similar results were obtained by Gordon et al. (2002) and Fedorenko et al. (2006) with a memory load paradigm and by Barker et al. (2001) with a sentence-completion task on agreement attraction. Hence, even though the effect was detected at the critical retrieval region (i.e., the verb), it must reflect encoding interference.

Additional findings pointing to the critical role of encoding interference in sentence comprehension have also been provided by Hofmeister and Vasishth (2014) in a self-paced reading study. In sentences in which the to-be-retrieved object (*the general*) was modified by an object relative clause (e.g., *The congressman interrogated the general who a lawyer for the White House advised to not comment on the prisoners*), the authors observed faster reading times at the verb (*advised*) when the target was semantically and syntactically complex (*the victorious four-star general*) as compared to when it was simple (*the general*). Again, the complexity of the target is not a retrieval cue, and the authors interpreted this finding as supporting encoding interference.

In two studies using a memory-load paradigm in a self-paced reading task, Kush et al. (2015) manipulated the words in the memory load such that they either rhymed or not with the to-be-retrieved element (*the boat*) in an object cleft clause (e.g., Rhyme Memory Load: *coat, vote, note*; No Rhyme Memory Load: *table, sink, truck*; Sentence: *It was the boat that the guy who drank some hot coffee sailed on two sunny days*). Reading times were longer at the second noun phrase region (*that the guy*) in the rhyme condition as compared to the no-rhyme condition, thus attesting to a detrimental effect of phonological overlap at encoding. Since no effect was observed at the critical verb region, the findings were taken as evidence that phonological features fail to affect retrieval processes, contra Acheson and MacDonald (2011) who interpreted phonological interference effects as attesting to retrieval interference. It’s interesting to note that studies by Gordon et al. (2001, 2002) also reported interference effects at the second noun phrase. However, as noted by Van Dyke and McElree, 2006, these effects were not unequivocally interpretable in terms of encoding interference (in Gordon et al., 2001, pronouns were both shorter and more frequent than definite descriptions, and in Gordon et al., 2002, the interference effect was already attested in the region containing the first noun phrase).

Although these studies provided evidence for encoding interference, a recent study by Jäger et al. (2015), designed to disentangle encoding and retrieval interference, concluded against the role of encoding interference in the processing of reflexive dependencies. In three experiments, the authors tested the effect of gender match between a target and a distractor in contexts in which the retrieval site contained no gender feature (i.e., the German reflexive, *sich*, and the Swedish reflexive possessive, *sin*, which are not gender marked) and in contexts in which gender was present at retrieval site (i.e., Swedish possessives, which are gender marked, *hans-M*). Results from the two German experiments (self-paced reading and eye-tracking) showed no on-line effects of gender match between the target antecedent and a distractor. However, an effect was found off-line, with higher accuracy rates in the gender mismatch condition. Since the German reflexive (*sich*) is gender neutral, the effect on accuracy is only compatible with encoding interference. Second, for Swedish possessives, both an on-line and an off-line mismatch effect were observed, while no effect was found for reflexive possessives. However, and surprisingly, the on-line effect found in possessives went in the opposite direction to what is predicted by the similarity-based interference hypothesis: more regressions were observed in the mismatch condition than in the match condition. To account for this unexpected result, the authors suggested that it reflected the misretrieval of the interfering element (and thus an erroneous interpretation of the sentence). Despite the fact that this assumption requires adjustments in the ACT-R model (Lewis and Vasishth, 2005) that, as such, does not predict misretrieval, the authors concluded in support of retrieval interference, putting aside the off-line German results supporting encoding interference. It is interesting to notice that off-line interference effects are not isolated. Gordon et al. (2001, 2002) as well as developmental studies (Adani et al., 2010, Adani, 2012; Belletti et al., 2012; Bentea et al., 2016; Adani, 2008, Unpublished) reported off-line interference effects, manifest in measures of sentence comprehension of object relative clauses (although it is unclear whether these effects lie at retrieval or at encoding).

### Aims of the Current Study

Although the prominent cue-based retrieval model of memory for sentence comprehension has granted a key role to similarity-based interference in target retrieval, closer inspection of existing evidence suggests that many of the observations taken as evidence for interference at retrieval are actually compatible with the hypothesis that interference arises at encoding. We have pinpointed a few studies providing conclusive evidence either for retrieval interference or for encoding interference. However, these studies were conducted on different long-distance dependencies, different types of features, different populations, and they also involve different measures (on-line vs. off-line). Moreover, with the exception of Franck et al. (2015), the adult literature on interference involving agreement features suggests that similarity in terms of these features plays no role in the comprehension of grammatical sentences; effects were indeed for the most part observed in ungrammatical sentences (e.g., Wagers et al., 2009; Dillon et al., 2013; Tanner et al., 2014; Lago et al., 2015; Tucker et al., 2015). This finding, entirely based on on-line measures, contrasts with off-line measures in children showing improved comprehension of object relatives when the object and the subject have different number or gender features.

In the present study, we collected both on-line and off-line measures in adults’ processing of strictly grammatical object relative clauses (ORs) in which we manipulated similarity between the object and the subject in terms of number and gender as well as the presence of an agreement retrieval cue at the verb. We did so by taking advantage of selective properties of Italian and English object relative clauses. In Italian, the verb never agrees with the subject in gender, therefore providing no gender cue for retrieval. In English, present tense verbs morphologically express number agreement with the subject (e.g., *criticizes-SG*), but past tense verbs do not (e.g., *criticized-Ø*). This design allowed us, first, to determine whether off-line effects of agreement features’ similarity found in children replicate in adults, and second, to determine whether these effects arise at retrieval, encoding or both:

(i) If interference affects only retrieval, a detrimental effect of feature match is expected in the present tense in English, but not in the past tense nor in Italian;^1^
(ii) If interference affects only encoding, a detrimental effect of match is expected in Italian as well as in English, where a similar effect is expected for present and past tense verbs;
(iii) If interference plays a role both at retrieval and at encoding, the detrimental effect of match should take the form of an interaction in English, with a small, but significant effect in the past tense, and a stronger effect in the present tense.

Anticipating the results, we observed clear effects of match in off-line accuracy measures, both in Italian and in English, replicating developmental data. Importantly, these effects were found independently of the presence of agreement retrieval cues on the verb, supporting the hypothesis that the locus of these interference effects is encoding. In line with previous adult data, on-line effects appeared much weaker; nevertheless, they seem non-negligible, and interestingly, they seem more pronounced when the verb carries an agreement retrieval cue (English present tense) than when it does not (English past tense). This suggests a role, though weak, of retrieval interference on-line. Overall, the robust effect of encoding interference reported here challenges cue-based retrieval memory models (such as ACT-R, Lewis and Vasishth, 2005) which fail to incorporate a mechanism for it. In the Section “General Discussion,” we propose a mechanism of activation leveling able to generate encoding interference in ACT-R. We argue that assuming two different mechanisms, accounting separately for encoding and retrieval interference, is non-parsimonious, and show how a self-organized sentence processing model allows accounting for them with a unified mechanism (Tabor and Hutchins, 2004).

## Experiment 1

### Materials and Methods

#### Participants

One hundred and sixty-seven participants took part in the experiment. Participants were all native speakers of Italian (mean age = 33 years old, *SD* = 9.48, age range = 16–69 years old) and they were all naïve to the purpose of the experiment. The laboratory-based experiment was approved by the ethics committee of the University of Geneva. For the on-line version, participants gave their consent to take part in the research prior to the beginning of the test by ticking a box in the online platform.

#### Materials and Design

Thirty-two sets of four conditions each were generated in a 2 × 2 design by manipulating: (i) the gender of the object (masculine vs. feminine), and (ii) the match between the gender of the subject and the gender of object (match vs. mismatch). Noun phrases were always animate and singular. The gender of nouns was expressed both on the determiner (e.g., *il-M/la-F*) and on the noun (e.g., *ballerin-o-M/ballerin-a-F*). The experimental items consisted of object relative clauses adapted from the sentences of a French experiment for which semantic reversibility was controlled (see Villata and Franck, 2016)^2^. All sentences were thus semantically reversible, so that it was not more likely for the agent to perform the action described by the verb than for the patient. In Italian relative clauses, the past participle (*sorpreso*) never agrees in gender with the subject, therefore remaining in its masculine default form.^3^ Examples of experimental items are presented in **Table 1**. Filler sentences consisted of complex sentences involving movement and/or subordination, and subject relatives. They were decomposed into a varying number of reading windows, depending on their length. Eight lists were created in order to reduce the number of experimental sentences participants were confronted with since filler sentences also contained relative clauses tested for the purpose of another experiment not reported here. Each participant was thus presented with 72 sentences in total, 16 experimental sentences and 56 filler sentences. Experimental sentences were decomposed into 11 regions.

**Table 1:** Example of item in the four experimental conditions of Experiment 1.

Experimental conditions	
**Masculine object**	
Match (MM)	Il/ballerino/che/il/cameriere/ha/sorpreso/beveva/un/cocktail/alcolico
	*The/dancer-MASC/that/the/waiter-MASC/has/surprised-Ø/drank/a/cocktail/with alcohol*
Mismatch (MF)	Il/ballerino/che/la/cameriera/ha/sorpreso/beveva/un/cocktail/alcolico
	*The/dancer-MASC/that/the/waiter-FEM/has/surprised-Ø/drank/a/cocktail/with alcohol*
**Feminine object**	
Match (FF)	La/ballerina/che/la/cameriera/ha/sorpreso/beveva/un/cocktail/alcolico
	*The/dancer-FEM/that/the/waiter-FEM/has/surprised-Ø/drank/a/cocktail/with alcohol*
Mismatch (FM)	La/ballerina/che/il/cameriere/ha/sorpreso/beveva/un/cocktail/alcolico
	*The/dancer-FEM/that/the/waiter-MASC/has/surprised-Ø/drank/a/cocktail/with alcohol*


#### Procedure

The experiment was programmed on Ibex Farm^4^ (Drummond, 2013), an online experimental javascript-based platform that uses the local machine for timing, thus achieving very accurate timing (see Crump et al., 2013; Enochson and Culbertson, 2015). Sentences were presented on a computer screen in a moving-window self-paced reading paradigm (Just et al., 1982): a series of dashes corresponding to the words of the sentence, with spaces between them, are presented on the screen, and as soon as the participant presses the space bar the first word appears, replacing the corresponding dashes. Subsequent button presses make each subsequent word appear, while the previous words disappear (non-cumulative presentation method). In our design, the items were presented in a random order. As soon as the last word of the sentence disappeared, a yes/no comprehension question was displayed at the center of the screen and participants were asked to answer the question by clicking with the mouse on one of the two available answers (yes vs. no). Comprehension questions always targeted thematic role attribution in the relative clause (e.g., *Did the waiter surprise the dancer?* vs. *Did the dancer surprise the waiter?*), thus allowing us to determine if the correct parse of the sentence was built. Instructions encouraged both rapid reading and correctness in answering the question. The experimental session began with four practice trials. The whole session lasted about 15 min.

### Results

#### Data Analyses

Reading times were analyzed with linear mixed-effects regression models (generalized linear mixed-effects regression models for the comprehension questions) using the lme4 package (Bates et al., 2015) in R (R Development Core Team, 2016). Only items for which the comprehension question was answered correctly were included in the analysis of reading times. Reading times greater than 3000 ms or less than 100 ms (which corresponds to 2.5 standard deviation from the mean by region and condition) were removed (affecting 2% of the data). No additional outlier removal process was performed. However, in a rapid visual serial presentation task, Staub (2010) showed that the effect of a mismatching intervening subject in object relative clauses was driven by a small set of trials, and in particular those trials that have disproportionately long reaction times (see also Lago et al., 2015 for similar results with a self-paced reading task). We thus conducted an additional analysis adopting a more conservative trimming, excluding only reading times exceeding 8000 ms (affecting less than 1% of the data) in case the occurrence of an effect depended on inclusion of the right tail of the reading time distribution. The 8000 ms cut-off point was chosen because it affected very few data points and removed only those data points that were very isolated form the others in visual inspection of the data.

Reading times were log-transformed to normalize residuals and then regressed against two factors that are known to affect reading times in self-paced reading tasks, namely word length and the log list position of the sentence in the stimuli (i.e., longer reading times are associated with longer words and faster reading times with later list position; Hofmeister, 2011; Hofmeister and Vasishth, 2014). The *residual log reading time* is therefore the dependent variable analyzed here. Error bars in graphs represent standard errors of the subject means.

All our predictive factors were dichotomous and centered by coding one level of the factor as -1 and the other as 1. We always used the maximal random-effects structure by participant and by item justified by the data. No correlations between random effects were estimated. Our analyses are therefore conservative with respect to the generalizability of the effects of theoretical interest to new participants and items (Barr et al., 2013). *P*-values were calculated by way of Satterthwaites’s approximation to degrees of freedom with the lmerTest package (Kuznetsova et al., 2015).

To assess the gender mismatch effect, we performed analyses on three separate regions: the critical region containing the past participle (region 7), the matrix verb region that follows it (region 8), and the region containing the second noun phrase (i.e., the subject, region 5). We analyzed the subject region to test the hypothesis that encoding effects might manifest at the point of encoding (Van Dyke and McElree, 2006).

#### Comprehension-Question Accuracy

Mean accuracy scores of question responses are provided in **Table 2**. Generalized linear mixed effect analysis revealed a significant main effect of gender match (β = -0.366, *SE* = 0.06, *z* = -5.636, *p* < 0.001) attesting to higher accuracy scores for mismatch conditions than match conditions. No other effect was significant (*t_s_* < 1).

**Table 2:** Mean accuracy percentages for comprehension questions by experimental condition in Experiment 1.

Condition	Accuracy	Standard deviation
Gender match, feminine object	75.5	0.42
Gender match, masculine object	77.9	0.41
Gender mismatch, feminine object	84.6	0.36
Gender mismatch, masculine object	83.9	0.36


#### Reading Times

**FIGURE 1:**
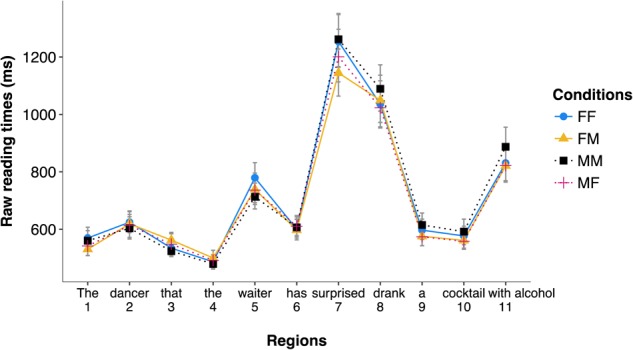
Distribution of reading times (in ms) in the four experimental conditions for the different regions of Experiment 1 (correct trials only).

The distribution of reading times across the four experimental conditions is reported in **Figure 1**. We plotted non-transformed reading times for readability, but analyses were conducted on residual log reading times.

*Region 7* (*surprised*). No effect was significant (*t_s_* < 2).
*Region 8* (*drank*). No effect was significant (*t_s_* < 1).
*Region 5* (*waiter*). No effect was significant (*t_s_* < 2).

In line with Staub (2010), we then go through a more conservative trimming, excluding only reading times exceeding 8000 ms. If the match effect depends on the right tail of the distribution, then it may show up when longer reading times are conserved in the analyses.

*Region 7* (*surprised*). Results attested to a significant effect of gender match (β = 0.031, *SE* = 0.014, *t* = 2.253, *p* = 0.025), with faster reading times for mismatch conditions (*M* = 1197 ms) than match conditions (*M* = 1316 ms). No other effect was significant (*t_s_* < 1).

*Region 8* (*drank*). No effect was significant (*t_s_* < 1).

*Region 5* (*waiter*). Results attested to a marginally significant effect of the gender of the object (β = -0.020, *SE* = 0.010, *t* = -1.921, *p* = 0.068), attesting to longer reading times at the second noun phrase region for feminine objects (*M* = 763 ms) than for masculine objects (*M* = 728 ms). Further models attested that this difference was entirely driven by the condition with two feminine noun phrases, which had marginally significant longer reading times as compared to the condition with two masculine noun phrases (β = -0.053, *SE* = 0.028, *t* = -1.901, *p* = 0.057), while all other conditions were on a par.

No other effect was significant (*t_s_* < 1).

### Discussion

Experiment 1 found a main effect of gender match in comprehension accuracy, such that sentences in gender mismatch conditions were understood better than those in match conditions, suggesting similarity-based interference. The same effect, though weaker, was found on-line, but only when a more conservative trimming was used, in which case reading times at the critical past participle region were faster in the gender mismatch conditions. This result is in line with findings on number agreement by Staub (2010) and Lago et al. (2015), who showed that the number mismatch effect in object relatives lay in the right tail of the distribution, i.e., was driven by slow trials. Thus, both the off-line and, to a lesser extent, on-line effects point to the role of encoding interference, since in Italian the past participle does not agree in gender with the subject and gender is therefore not a retrieval cue. In the Section “General Discussion,” we consider two models which both predict encoding interference effects at the verb: the interference makes the distractor a stronger competitor for attachment to the verb.

It has been argued that encoding interference should also manifest immediately at the region in which an element similar to a previously encoded element is encountered (see Van Dyke and McElree, 2006). This prediction is not borne out by our results, since we found no difference between mismatch and match conditions at the subject region. In fact, evidence for interference at the second noun phrase is scarce in the literature: only two studies have reported clear evidence for such an effect (Acheson and MacDonald, 2011; Kush et al., 2015). We will discuss a possible reason why encoding interference does not already manifest when the interfering noun phrase is encountered in on-line results in the Section “General Discussion,” after having established whether it replicates in Experiment 2.

Results from Experiment 1, showing evidence for a facilitatory effect of gender mismatch in the comprehension of Italian object relatives, stand in contrast with results from the developmental study of Belletti et al. (2012). The authors found no effect of gender mismatch in the comprehension of object relatives in Italian-speaking children, although they found a significant effect in Hebrew-speaking children, a language in which gender is marked on the verb. However, Belletti et al. (2012) Italian data exhibited a clear numerical tendency toward mismatch facilitation (*M* = 57% vs. *M* = 52%; *p* = 0.16 in the ANOVA by subjects and *p* = 0.14 in the ANOVA by items). Our results therefore suggest that the null result on which Belletti et al. (2012) capitalized is actually a Type II error due to lack of power.^5^

Although most of the literature on agreement in sentence comprehension has failed to find any on-line effect of feature mismatch in grammatical sentences, our finding aligns with other data reported in French (Franck et al., 2015; Villata and Franck, 2016). We therefore suggest that the lack of a match effect in grammatical sentences reported in these studies is also a Type II error, due to design weakness and possibly to the smaller sample size tested in these studies as compared to our (Wagers et al., 2009; Dillon et al., 2013; Tanner et al., 2014; Lago et al., 2015; Tucker et al., 2015). If these studies had included an off-line measure, we believe that they would also have revelealed the effect found here. We will discuss a possible cause for the difference between the strength of on-line and off-line measures in the Section “General Discussion.”

Finally, our results also revealed a tendency toward longer reading times for feminine noun phrases than masculine ones. We hypothesize that this reflects the cost associated with the encoding of a marked feature (feminine) as compared to an unmarked one (masculine). Similar effects have also been attested in French for gender, where feminine noun phrases took longer to be encoded than masculine noun phrases (Villata and Franck, 2016) and in English for number, where a plural feature on the noun has a cost that spills over onto the next reading regions (Wagers et al., 2009).

To summarize, results from Experiment 1 provide support for encoding interference in Italian, where a gender mismatch facilitatory effect was observed even though gender is not a retrieval cue on the verb. We now turn to Experiment 2 which allowed us to contrast, within the same language (English), the presence vs. absence of an agreement cue on the verb in order to assess the possibility that both encoding and retrieval interference play a role.

## Experiment 2

### Materials and Methods

#### Participants

One hundred and thirty participants took part in the experiment. They were all undergraduates students at the University of Connecticut and they received course credit for their participation (ages generally in 18–22 years)^6^. They were all native English speakers and naïve about the purpose of the experiment. The experiment was approved by the ethics board of the University of Connecticut and informed consent was obtained from all participants.

#### Materials and Design

Thirty-two sets of four conditions each were generated in a fully crossed 2 × 2 design. We manipulated: (i) the match between the number of the subject and the number of the object (match vs. mismatch); since the subject was always singular, this amounted to manipulating the number of the object, and (ii) the presence of an agreement cue on the verb (cue, i.e., present tense verb, vs. no cue, i.e., past tense verb). The presence of an agreement cue on the relative verb was manipulated by taking either present tense verbs (e.g., *criticizes-SG*), which exhibit subject-verb number agreement, or past tense verbs, in which agreement is not morphologically expressed on the verb (e.g., *criticized-Ø*). Sentences were all object relative clauses with animate subjects and objects. As in Experiment 1, the experimental sentences were an adaptation of the experimental sentences used by Villata and Franck (2016) in French, for which thematic roles reversibility was controlled for. Therefore, no semantic cue was available for assigning thematic roles. Examples of experimental items are presented in **Table 3**. Verbal agreement was manipulated on the verb of the relative clause, while the matrix verb was kept in the past form in order to restrict the agreement cues present in the sentence. In order to control for potential spillover effects, which manifest when the reading times measured in region *n* are influenced by reading times in region *n-1*, an adverb was introduced before the critical region of interest (i.e., the relative verb). The critical verb (*criticize-s/-d*) was always followed by a complex quantifier phrase (e.g., *most of*) with a temporal modifier as complement (e.g., *the time*) followed by the matrix verb. As for Experiment 1, filler sentences consisted of complex sentences with movement and/or subordination and subject relatives. Eight lists were again created to reduce the number of relative clauses each participant encountered, since filler sentences also contained relative clauses tested for the purpose of another experiment not reported here. Each participant was presented with 72 sentences in total: 16 experimental sentences and 56 filler sentences. Experimental sentences were decomposed into a number of reading windows varying between 15 and 17, each containing either a content word or a grammatical word. Filler sentences were decomposed into a varying number of reading windows, depending on their length.

**Table 3:** Example of item in the four experimental conditions of Experiment 2.

Experimental conditions	
**Agreement cue**	
Match (SS)	*The/dancer-SG/that/the/waiter-SG/strongly/criticizes-SG/most/of/the/time/ordered/a/rum/cocktail.*
Mismatch (PS)	*The/dancers-PL/that/the/waiter-SG/strongly/criticizes-SG/most/of/the/time/ordered/a/rum/cocktail.*
**No agreement cue**	
Match (SS)	*The/dancer-SG/that/the/waiter-SG/strongly/criticized-Ø/most/of/the/time/ordered/a/rum/cocktail.*
Mismatch (PS)	*The/dancers-PL/that/the/waiter-SG/strongly/criticized-Ø/most/of/the/time/ordered/a/rum/cocktail.*


#### Procedure

The procedure was the same as Experiment 1, except that each word was presented in the center of the screen (centered, non-cumulative presentation). The experiment was programmed with the E-prime software (Schneider et al., 2012). Each trial began with a fixation cross (400 ms) followed by an interstimulus blank screen (150 ms) and then the word-by-word presentation of the sentence. Each trial was separated from the next by an instruction in which we asked participants to press the space bar as soon as they were ready to continue. After each sentence a yes/no comprehension question was presented on the computer screen. An interstimulus blank screen (150 ms) separated each sentence from the corresponding comprehension question, which appeared at the center of the screen. Comprehension questions specifically targeted the critical relative verb to determine whether the correct parse was built (e.g., *Did the waiter criticize the dancer?* vs. *Did the dancer criticize the waiter?*).

Instructions encouraged both rapid reading and correctness in answering the question. Items were presented in a fixed pseudo-random order constrained such that no more than two consecutive trials were experimental sentences.

Each experimental session began with four practice trials. Three breaks of 1-min each were administered during the task. The whole session lasted about 20 min.

### Results

#### Data Analyses

The same data analyses conducted for Experiment 1 were used here. To assess the number mismatch effect and its interaction with the presence of number cue at the verb, we performed analyses on three separate regions: the critical embedded verb region (*criticize-s/d*, region 7), the region immediately following the embedded verb to investigate potential spillover effects (region 8), and the region containing the second noun phrase (i.e., the subject, region 5) where an interference effect is expected to show up if the encoding process itself is affected by similarity (Van Dyke and McElree, 2006).

The error rate in comprehension accuracy for object relatives was particularly high (30% incorrect responses). However, the near ceiling performance in comprehension questions for filler items (95% correct responses) suggests that the high error rate in object relatives was not due to a general lack of attention during the task, but rather reflects a genuine difficulty in the processing of experimental sentences. Because we were interested in investigating the effectiveness of number cues in driving structure building, we restricted our investigation to items for which participants built the correct parse thus leading to a correct response.

#### Comprehension Question Accuracy

Mean accuracy scores of question responses are provided in **Table 4**. Generalized linear mixed effect analysis revealed a marginal effect of number mismatch (β = -0.088, *SE* = 0.051, *z* = -1.713, *p* = 0.086), attesting to numerically higher comprehension accuracy for number mismatch than number match conditions. No other effect was significant (*t_s_* < 1).

**Table 4:** Mean accuracy scores of question responses in percentage by experimental condition in Experiment 2.

Condition	Accuracy	Standard deviation
Number match, agreement cue	64.1	0.48
Number match, no agreement cue	65	0.47
Number mismatch, agreement cue	66.5	0.47
Number mismatch, no agreement cue	68.6	0.46


#### Reading Times

**FIGURE 2:**
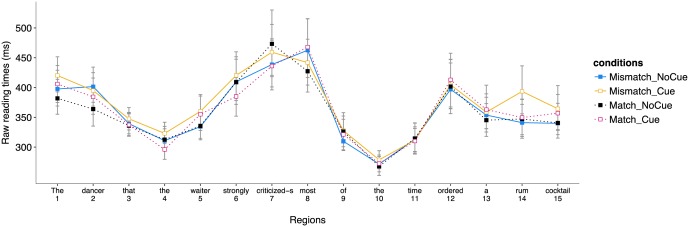
Distribution of reading times (in ms) in the four experimental conditions for the different regions of Experiment 2 (correct trials only).

The distribution of reading times across the four experimental conditions in correct trials is reported in **Figure 2**.^7^

*Region 7* (*criticized-s*). No effect was significant (*t_s_* < 1).
*Region 8* (*most*). No effect was significant (*t_s_* < 2).
*Region 5* (*waiter*). No effect was significant (*t_s_* < 2).

As for Experiment 1, in order to investigate whether the effect was in the right tail of the distribution, we conducted an additional analysis with a more conservative trimming in which only reading times longer than 8000 ms were eliminated.

*Region 7* (*criticized-s*). No effect was significant (*t_s_* < 2).

*Region 8* (*most*). A marginally significant interaction between the number match and the presence of an agreement cue on the verb was found (β = -0.026, *SE* = 0.015, *t* = -1.888, *p* = 0.059), revealing a tendency for an effect of number match in the agreement cue condition (β = 0.072, *SE* = 0.040, *t* = 1.794, *p* = 0.073), with faster reading times at the spillover region in the number mismatch condition (*M* = 488 ms) than in the number match condition (*M* = 562 ms), but no effect was observed in the no agreement cue condition (*t* < 1).

*Region 5* (*waiter*). No effect was significant (*t_s_* < 1).

### Discussion

Results from Experiment 2 revealed a tendency for higher comprehension accuracy for mismatch than match conditions, an effect that did not interact with the presence of a number retrieval cue on the verb. The lack of interaction is in line with the hypothesis that similarity in number features affects encoding only, since the effect of feature mismatch is not modulated by the presence of a number retrieval cue at the verb. This conclusion is in line with results from Experiment 1. The fact that, unlike Experiment 1, the accuracy mismatch effect in Experiment 2 is only marginally significant is possibly due to the scarcity of English number agreement morphology. In particular, in present tense verbs only the third singular person subject has a distinctive number suffix; in the past tense, only the copula/auxiliary shows two forms, *was* and *were*, distinguishing the first and third singular persons from all other persons, while all other verbs have the same form for all persons, which may have reduced comprehenders’ reliance on agreement cues (MacWhinney and Bates, 1989). Moreover, English sentences were more complex than the Italian ones, since they involved a complex quantifier phrase with a temporal modifier as complement. The overall lower accuracy of the English sentences may have also contributed to reducing the chances of observing an effect of number mismatch. Nevertheless, it is notable that the observed tendency goes in the expected direction, showing numerically higher accuracy for number mismatch conditions.

As in Experiment 1, a weak online tendency toward faster reading times in conditions of feature mismatch was found in analyses that included longer reading times, thus suggesting that the effect is again tied to the right tail of the distribution. In contrast to Experiment 1, the effect did not appear at the critical verb region but in the region immediately following it. Interestingly, this effect was only found in the present tense condition, when an agreement retrieval cue was present on the verb. Since the match effect was modulated by the presence of a retrieval cue, this result provides some indication of an effect of match arising at retrieval. However, the absence of a facilitatory mismatch effect in the past tense condition is at odds with the Italian results that show a significant online mismatch effect in the absence of a retrieval cue on the verb. We tentatively suggest that the absence of an effect in the English past tense condition may also be a consequence of the poor agreement morphology of English: the effect of number mismatch may be even more difficult to detect when no agreement feature is present on the verb. That is, the mismatch effect would be stronger in Italian due to the rich morphology of that language, such that the effect manifests more clearly than in English both off-line and on-line, and it manifests despite the lack of morphological cue on the verb, while in English morphological marking at the verb is necessary for the effect to show up.

All in all, results from Experiment 2 provide evidence, although weak, for a facilitatory number mismatch effect in the comprehension accuracy of grammatical sentences, extending to English results from Experiment 1 in Italian, as well as results in French (Franck et al., 2015, Experiment 1; Villata and Franck, 2016), and for a facilitatory number mismatch effect in the agreement cue condition in online measures, providing evidence, for retrieval interference. This evidence is weak in the sense that the effect was only detected when longer trials are included in the analyses.

## General Discussion

### Summary of the Findings

We conducted two self-paced reading experiments designed to explore interference due to agreement feature similarity in sentence comprehension, as well as the locus of this effect: encoding, retrieval, or both. We tested the effect of gender and number similarity between a subject and an object in the comprehension of object relative clauses in Italian and English. In Italian, the verb never agrees in gender with its subject; therefore gender provides no cue for subject retrieval. In English, the verb is morphologically marked for number agreement in the present tense, thus providing a subject retrieval cue, but not in the past tense. We reasoned that if a match in agreement features affects encoding only, then a facilitatory mismatch effect would be expected independently of whether the verb carries an agreement marker or not, and should therefore be found across the board. If feature match affects retrieval only, then a facilitatory effect of mismatch should emerge in the English present tense condition only, the only case in which retrieval cues were marked on the verb. Finally, if both encoding and retrieval interference are at play, a facilitatory mismatch effect should show up in Italian as well as in both conditions in English, but it should be stronger in the present tense than in the past tense.

In line with the hypothesis that agreement feature similarity affects encoding, we found that: (a) feature similarity affects off-line comprehension accuracy, with feature match causing more errors of comprehension than feature mismatch, even when the verb carried no agreement cue (significant effect in Italian, marginal effect in English); (b) feature similarity weakly affects on-line processing of the verb (Italian), an effect that is observed only when long trials (over 3000 ms) are included (significant effect). In line with the hypothesis that agreement feature similarity also affects retrieval, we found that a weak on-line effect of feature similarity in English was only found in the present tense condition, when number provided a retrieval cue on the verb, while no effect was found when the verb carried no agreement marker (marginal interaction). To account for results from Experiments 1 and 2 we thus need a model generating both encoding and retrieval interference. In Sections “An Encoding Interference Mechanism in ACT-R: Activation Leveling” and Section “A Self-Organized Sentence Processing (SOSP) Account of Encoding and Retrieval Interference Effects” we discuss two alternative models that do this.

All in all, our finding that similarity in agreement features affects the comprehension of grammatical sentences in Italian and English aligns with other adult studies – Franck et al. (2015) and Villata and Franck (2016) in French, Jäger et al. (2015) in German and Swedish – as well as developmental studies in Italian (Adani et al., 2010), English (Adani, 2012), Hebrew (Belletti et al., 2012) and German (Adani, 2012), which all provide evidence for encoding interference effects. Similarity-based interference in all these studies manifested in off-line measures of comprehension accuracy (with the exception of Franck et al., 2015 who found an effect on-line). The weak effect reported on-line in Experiments 1 and 2, despite the large sample sizes, is also compatible with the lack of effect reported in most on-line studies (e.g., Wagers et al., 2009; Dillon et al., 2013; Lago et al., 2015), and suggests that the lack of significance in these studies is due to the weakness of the effect. If the interference effect is more robust offline, as at least our Italian results suggest, these studies may simply not allowing us to detect such an effect. Moreover, these prior relevant studies have all been conducted in English. If, as suggested above, agreement cues are weaker in English due to their low availability in the input (MacWhinney and Bates, 1989), we cannot exclude that the absence of an effect of number mismatch in prior studies on English grammatical sentences might be due to the morphological specificity of that language.

Based on these findings, we now consider parsing mechanisms that predict both encoding and retrieval effects. First, we describe a way that a cue-based retrieval memory model like ACT-R (Lewis and Vasishth, 2005) can be augmented to account for the encoding effects we have observed. We then explain how a parsing model based on self-organization (Kempen and Vosse, 1989; Vosse and Kempen, 2000; Tabor and Hutchins, 2004; Van der Velde and de Kamps, 2006; Smith et al., in press), implements retrieval and encoding interference, and arguing that unlike ACT-R, the self-organizing approach naturally captures encoding interference effects a consequence of its main structure-building mechanism, and hence offers a more parsimonious explanation.^8^

### An Encoding Interference Mechanism in ACT-R: Activation Leveling

In the Section “Introduction,” we noted that prior researchers have suggested that one possible mechanism underlying encoding interference is *feature overwriting* (Nairne, 1990; Oberauer and Kliegl, 2006). Feature overwriting is a mechanism in which, at the point of encoding, if two arguments share a feature, they enter in a competition for the shared feature (under the hypothesis that features are represented as unique units, which cannot therefore belonging to more than one item at the time) and the element losing the competition is supposed to lose the feature, thus resulting in a degraded mental representation. Since feature overwriting only occurs in match conditions (i.e., conditions in which the two elements share one feature), it correctly predicts encoding effects to arise in these conditions only.

Vasishth et al. (2017) argued that a statistical model implementing feature overwriting provides a better fit to account for the difficulty associated with a match in agreement features than a statistical model implementing a cue-based retrieval account. In particular, the authors tested a critical assumption of the feature overwriting hypothesis, namely that feature overwriting mostly occurs in conditions of feature match, and to a lesser extent in conditions of feature mismatch (Nairne, 1990). This implies that reading times for both match and mismatch conditions derive from two different distributions: the distribution in which no feature overwriting has occurred (which would have a certain mean and a certain standard deviation) and the distribution in which feature overwriting has occurred (whose mean and standard deviation will be larger), unlike the cue-based retrieval interference account, which does not assume a mix of distributions. Results from a Bayesian hierarchical two-mixture model supported this assumption: when feature overwriting has occurred (match condition), the model that best characterizes the data is the one assuming that a proportion of trials comes from a distribution with larger mean and standard deviation, in line with the feature overwriting hypothesis and contra the cue-based retrieval interference model.

However, feature overwriting alone does not specify how parsing works, so we now take up the question of how encoding interference could be generated in a parsing model, like ACT-R, which is the most highly developed model of interference effects in sentence processing. Although feature overwriting causes featural changes at encoding, if implemented as such in ACT-R or another cue-based retrieval frameworks, it would have no effect at retrieval whenever the overlapping feature was not a retrieval cue. This is the case for the Italian and English past tense conditions here, as well as for several studies in the literature that found encoding interference at retrieval even in the absence of a retrieval cue (Gordon et al., 2001, 2004; Hofmeister and Vasishth, 2014). We therefore consider another way of generating encoding interference effects which fits naturally into the framework of ACT-R.

In standard ACT-R, what it is retrieved is a chunk, i.e., a feature bundle that can enter into a relation with other chunks (see Lewis and Vasishth, 2005). Chunks are retrieved based on their activation level, which is determined by: (i) the base activation level of the chunk, which is, in turn, determined by the past activations and reactivations of the chunk, and by the time elapsed since the last reactivation because of decay, (ii) the strength of the association between each retrieval cue and the chunk, that is the uniqueness with which the cue identifies the chunk (*fan effect*) and (iii) a random noise component. In ACT-R, retrieval functions in a “winner-take-all” race fashion: a chunk has to reach a fixed, high threshold of activation in order to be selected as a target of retrieval, and the chunk that reaches the threshold of activation first is retrieved. As a result, conditions in which a feature is spread across two or more chunks (match) have slower retrieval than conditions in which the feature is unique to a chunk (mismatch) (*fan effect*). Since competition effects in ACT-R are treated as competition for activation, it is natural to assume that featural similarity at encoding produces activation competition. To produce this effect, we add the further assumption (iv) that when a new chunk shares a feature with one (or more) element that has already been encoded, the activations of all chunks sharing that feature become more equal. We call this the *leveling effect*. Thus, if two elements are not already equal in activation, the higher one goes down and the lower one goes up. In our stimuli, when the parser encounters the subject noun phrase, a highly activated trace of this noun phrase is created. This subject trace is more activated than the object trace because it has been recently activated, while the object will not be reactivated until the object trace is encountered. Therefore, in our stimuli, leveling has the effect of reducing the subject’s activation and increasing the activations of other chunks. The current proposal thus extends the principle of activation-sharing that is present in the fan-mechanism to all features that are shared across chunks, whether they are retrieval cues or not.^9^

Since the leveling mechanism applies to all features, including features that are retrieval cues, one might consider eliminating the fan mechanism in favor of leveling alone (thus treating what have been viewed as retrieval interference effects as encoding interference effects). However, our English on-line results suggest (weakly) that retrieval cues at the verb enhance the effect of agreement feature similarity and, as we noted, the results of Van Dyke and McElree, 2006 and Belletti et al. (2012) are not predicted by an encoding-interference-only model. Therefore, we propose to have both fan and leveling in the model.

This model generates all the effects reported in the present paper. First, the model generates the on-line agreement feature match effect (match slower than mismatch) observed in Italian when no agreement cue was present at the verb, as well as in the English present tense condition when a cue was present, by lowering the activation of the candidate leading the activation race (i.e., the subject), so the race takes longer to conclude. Second, the model generates the agreement feature match effect in off-line comprehension questions because reducing the level of activation of the subject reduces the chances that it is correctly selected, and thus that the sentence structure is correctly built. Moreover, as we anticipated above, the model generates the on-line interaction (marginally significant in the data) between agreement feature similarity and the presence of an agreement cue on the verb in English because, in the present tense condition, fan favors the mismatch over the match condition, over and above the effects of leveling.

The addition of fan and leveling to ACT-R is a natural way to get the original ACT-R framework, which does not predict encoding effects, to predict them. However, it would be theoretically more parsimonious to derive the observed encoding interference effects from independently needed assumptions. In the last section, we discuss a self-organizing sentence processing framework in which such effects follow from independently motivated assumptions about the core structure-building processes that support sentence processing.

### A Self-Organized Sentence Processing (SOSP) Account of Encoding and Retrieval Interference Effects

Information transfer in ACT-R is unidirectional and discrete: newly arriving chunks search preceding chunks for cues that specify an optimal attachment locus (forward information flow). Then an instantaneous attachment choice is made. By contrast, in self-organized sentence processing (SOSP; see Kempen and Vosse, 1989; Vosse and Kempen, 2000; Tabor and Hutchins, 2004; Van der Velde and de Kamps, 2006; Smith et al., in press) information flow is multi-directional and continuous: attachments are made at any time during structure building. Moreover, the multi-directional, continuous flow is central to the way the model builds structure. We explain next how this flow takes circumstances that start out with mere similarity of encoding and turns them into situations of retrieval interference. Thus, under SOSP, encoding and retrieval interference stem from the same mechanism.

In SOSP, each perception of a word activates a treelet (a mother node with a finite set of daughter nodes) in memory similar to a “chunk” in ACT-R (see also Fodor, 1998a,b, 2017; Marcus, 2001). As activated treelets accumulate in memory, they attempt to combine in all possible ways with other activated treelets, subject to the restriction that daughter nodes only attempt to link with mother nodes of other treelets (no daughter-to-daughter or mother-to-mother connections and no within-treelet connections). Each mother and each daughter is a vector of semantic and syntactic features encoding the properties of the attachment site; treelets with multiple daughters make the daughters available for attachment in series, reflecting word-order constraints. All links start out, at the beginning of processing, with strength 0, and the strengths are constrained to lie in the interval [0, 1]. Each link strength grows and/or shrinks over time following two principles: (a) the better the match between the feature vectors at its ends, the more rapidly the link strength grows, and (b) links for the same attachment site compete so that, ultimately, one link wins each competition. This leads, most of the time, to the formation of a well-formed syntactic tree structure. Furthermore, the numerical values are noisy so there is some variation in how precisely the system adheres to principles (a) and (b). Finally, within the constraints imposed by the semantic and grammatical requirements of a treelet, the feature vectors on opposite ends of a link migrate toward the same values as the link grows stronger. Since this is happening among all activated treelets at once and continuously, information flow is multi-directional. In self-paced reading (and also language production – see below) the model moves to the next word when the attachments for the current word have nearly stabilized. After a sentence has been processed, the link strengths return to 0, but the treelets linger in the states they have gravitated to under the feature passing. This supports immediate reconstruction of the tree for purposes of repeating the sentence or answering questions about it.

To illustrate how this system generates encoding interference effects, we first describe its operation in a case related to the cases we report here. In the standard preamble continuation paradigm that Bock and Miller (1991) used to study agreement attraction, Barker et al. (2001) found that participants were more likely to produce a plural verb in examples like (2a), where the subject head and the attractor share a fine-grained semantic feature (boat-hood) than (2b), where they do not.

(2a) The canoe near the sailboats…
(2b) The canoe near the cabins…

**FIGURE 3:**
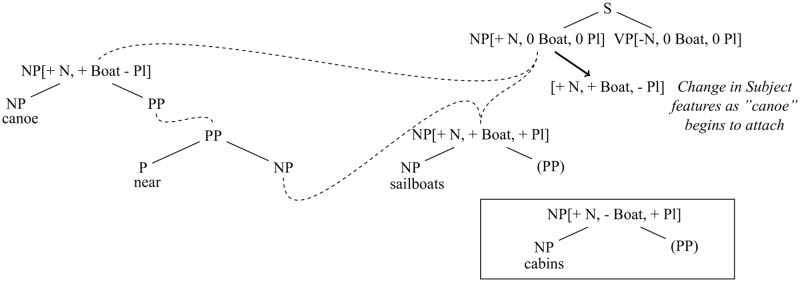
As the “canoe” treelet begins to attach to the NP (Subject Node) of S, the features of this node gravitate toward <+NP, + Boat, –Plural>. Consequently, when “sailboats” arrives, it matches the Subject Node on two features (+NP and +Boat) and competes strongly for attachment as subject, pushing the Plural feature of the Subject Node toward +. By contrast, when “cabins” arrives, it matches the Subject Node on only one feature and therefore does not push the Plural feature as strongly toward +. (Note: The VP Node of S is constrained to have the same Plural value as the Subject Node. Therefore, the state of the Subject Node determines the choice of verb number).

Such effects are not predicted by cue-based retrieval accounts, even when a cue-based retrieval model is extended to handle production (e.g., Badecker and Kuminiak (2007) propose such an extension of ACT-R for agreement attraction in Slovak). This is because, in those models, the property of being a boat is not relevant to the selection of the form of the verb. By contrast, in SOSP, when the first two words of the preamble have been spoken (see **Figure 3**), a treelet is activated by “canoe” that has features on its mother including <+Noun, +Boat, -Plural>. This treelet begins to combine with a verb treelet (whose lexical anchor has not yet been generated) and causes the subject daughter of the verb, which is already specified to be <+Noun>, to gravitate toward a feature vector that includes <+Noun, +Boat, -Plural> (the gravitation is a gradual process in which features originally set to the value 0 continuously increase their values toward a maximum of 1, driven by the growing strength of the link between the “canoe” treelet and the subject daughter of the verb). This feature convergence mechanism is, in effect, a form of *feature passing*.^10^ While this is happening, treelets for “near,” “the,” and “sailboats” are activated. The “sailboats” treelet bears the features <+Noun, +Boat, +Plural>. Because of the growing presence of the feature <+Boat> on the verb treelet, there is an enhanced tendency for the “sailboats” treelet to form a link with the subject daughter of the verb because “sailboats” also has the feature <+Boat>. This possibility of erroneously attaching the “sailboats” treelet as the subject of the verb follows from the assumption that all possible combinations attempt to form. During its interaction with the subject site, “sailboats” can cause the number feature of the subject daughter to gravitate to <+Plural>. This can happen even though “canoe” ultimately wins the competition to attach as the subject of the verb. When it is time to generate the verb, the model produces the verb that is most highly activated within the verb treelet. Consequently, in Barker et al.’s design, there is a greater chance that a plural verb will be produced after “the canoe near the sailboats” than after “the canoe near the cabins,” since in the latter case, “cabins” does not have the feature <+Boat> and therefore its treelet is less prone to being momentarily attached as the subject of the verb. It is the passing of features from “canoe” to the verb followed by the interaction of “sailboats” with the verb before a decision has been made about the choice of verb state that allows for interference (i.e., multi-directional information flow, combined with continuity of attachment, supports the generation of encoding interference effects). Moreover, once the features have become even slightly transferred from “canoe” to the verb, the situation turns from a pure encoding-similarly scenario into a retrieval interference scenario.

Turning now to the present experiments, when the embedded relative verb is processed, the verb treelet makes an attachment site available for its subject and one for its object. As the attachment between the embedded subject and the subject slot on the verb begins to form, the subject’s features, including its agreement features, are gradually transferred to the subject slot on the verb (as in the Barker et al. example above), while a similar mechanism takes place as the extracted object starts to attach to the object slot. Since all treelets compete for attachment at all sites, there is subject-object competition: the extracted object also attempts to attach as the subject of the embedded verb and the embedded subject attempts to attach as the object of the embedded verb.^11^ The intensity of this competition is stronger if the embedded subject and extracted object are featurally more similar (match condition), because this makes each of them fit the other’s slots on the verb better. Since the processor waits to continue until this attachment decision has been relatively resolved, the match condition produces longer processing times than the mismatch condition. However, this effect is weak at the beginning because the features have only been weakly transferred to the verb treelet (feature passing is a gradual transfer). Over time, the effect of the feature passing becomes stronger and, as a result, the effect of match becomes stronger. The dynamics of feature passing is thus responsible for two aspects of the effects of match reported in our two experiments: the fact that the on-line effect is only statistically reliable when slow trials are taken into account, and the fact that the effect becomes even stronger when a later measure is taken, as is the case of off-line responses.^12^ The model also explains our on-line finding in Experiment 2 that feature match has a stronger role when the verb is marked for number: if the verb is marked for agreement, it endows its subject slot with an agreement feature that uniquely matches that of the embedded subject only in the mismatch condition, which strengthens the correct link between the subject and the verb (in contrast to the match condition in which the feature on the subject slot of the verb also matches that of the object).^13^ Finally, we also want to briefly comment on how SOSP captures the grammatical-ungrammatical asymmetry reported in the literature (e.g., Wagers et al., 2009). In grammatical sentences, there is a snow-balling feedback effect because all constraints work together to powerfully and quickly drive formation of the correct parse. In the face of this force, potentially distracting elements have only a weak influence so little interference is detected. This is in line with the weak effect reported in the studies presented here, as well as with the null effect reported in many studies in the literature, which might have not attained enough power to detect such a small effect (e.g., Wagers et al., 2009; Lago et al., 2015). In ungrammatical sentences, there is no way to perfectly satisfy all the constraints so the system remains in a blended, intermediate state. The best thing going, in this case, is the ungrammatical match that is available just in the mismatch condition, so the mismatch condition thrives more than the match does (even though neither works completely well).

A key property of the self-organization approach is that it does not posit two different competition mechanisms for encoding and retrieval. Rather, the two types of effects stem from two different structural interactions: (1) encoding interference effects stem from the interaction between the two NPs: when the NPs are encountered and have to be attached to their slots on the verb treelet, the similarity in their agreement features plays a role in attracting each to the other’s attachment slot, and (2) retrieval interference effects stem from the interaction between the NPs and the verb: when the verb is encountered, its agreement feature attracts NPs that have the same agreement feature to its subject slot.

## Conclusion

In two self-paced reading experiments conducted in Italian and English, we have observed strong evidence for encoding interference, and weaker evidence for retrieval interference. We have proposed to implement an encoding mechanism in ACT-R, which in its current formulation does not predict encoding interference effects, by adding a mechanism of Activation Leveling which makes the activation of elements sharing a feature more equal. Equipped with this additional mechanism, ACT-R successfully accounts for both encoding and retrieval interference effects. Then, we showed how SOSP can also account for the results at hand. However, whereas ACT-R needs two separate mechanisms to handle retrieval and encoding effects (fan and leveling), under SOSP, a single computational mechanism generates both effects. This mechanism is competitive link formation with bidirectional information flow across links. It thus seems that SOSP is simpler than ACT-R, not only because it uses one mechanism to account for both encoding and retrieval interference rather than two, but also because this mechanism is the core process of hierarchical structure formation, independently motivated by the need to form parses.

## Author Contributions

SV, WT, and JF designed the experiments. SV and WT prepared the items. SV recruited participants and conducted all the studies reported. SV analyzed the data. SV, WT, and JF prepared the final manuscript.

## Conflict of Interest Statement

The authors declare that the research was conducted in the absence of any commercial or financial relationships that could be construed as a potential conflict of interest.

## Footnotes

1 By match and mismatch effect we always refer to the feature mismatch between the subject and the object of the relative clause.

2 Word length was controlled in statistical analyses (the log-transformed data were regressed against word length). The frequency of the verb (our critical region of interest) was controlled for in the French experiment from which the materials of this experiment were adapted. The frequency of the verbs in French was controlled through Lexique3 (New et al., 2001) and was based upon the frequency in subtitles in movies (which are closer to spontaneous speech). Frequencies in Lexique3 range from 0 to approximately 26000 per million words. We used verbs ranging in frequency from 7 to 126 per millions words (mean frequency = 54).

3 In Italian, past participle agreement holds with (i) unaccusative verb, (ii) passive morphology, (iii) direct object cliticization, (iv) reflexive clitics, and (v) impersonal passive *si* (see Belletti, 2006 for a discussion).

4 http://spellout.net/ibexfarm

5 This result has important consequences for syntactic theories of intervention like Relativized Minimality (Rizzi, 1990; Friedmann et al., 2009). Relativized Minimality assumes that only features that play an active morphosyntactic role (i.e., features that are syntactic attractors for movement) play a role in the computation of the similarity. But if a gender match effect is attested in Italian, in which gender does not belong to the set of features triggering movement, this undermines the authors’ conclusion that only features triggering movement play a role in the generation of interference effects see Villata and Franck (2016) for discussion.

6 Since Experiment 2 was carried out at the University and only undergraduates students took part in it, we did not collect more fine-grained information about participants’ ages.

7 Given the high rate of errors in comprehension, we also conducted analyses on the complete data set (both correct and incorrect trials). None of the models gave a significant results. Similar analyses were also conducted for Experiment 1 and none of the results was significant either. In fact, SOSP predicts an asymmetry in detection power in the observed direction (i.e., significant results on models conducted on significant trials only): because the SOSP activations are infused with noise, different trials of the same item do not behave identically. In general, mismatch enhances distinction between the subject and object sites on the verb. But the trials where it fails to do this will tend to be precisely those in which reconstruction for the comprehension question fails, i.e., the inaccurate trials. Moreover, when noise causes conflation of the competitors in mismatch conditions, it makes those conditions more like the match conditions, including causing them to have more elevated reading times. Thus, one expects the inaccurate trials to show a weaker reading time effect; including them in the analysis thus reduces the statistical power.

8 Another important mechanism in the agreement attraction literature is feature percolation. According to the percolation model (Vigliocco and Nicol, 1998; Franck et al., 2002; Eberhard et al., 2005), percolation takes place within the subject noun phrase. This model is thus inadequate to account for object attraction (we refer the reader to Wagers et al., 2009 for detailed arguments against what seems like the most natural extension of the percolation account to handle interference effects in object relative clauses). Moreover, the percolation of features from one noun phrase to another would not predict encoding interference effects since percolation happens independently of the featural similarity of the noun phrases.

9 This proposal bears some similarity to the RACE/R extension of ACT-R proposed by van Maanen et al. (2009) to capture encoding effects in domains other than sentence processing. To the three standard assumptions listed above, RACE-R adds the assumption that similar chunks mutually activate each another in proportion to the degree of their similarity. This correctly predicts poorer comprehension under match (because competitors are made proportionally more equal), but it wrongly predicts *decreased* reaction times under match (because both competitors are driven more quickly to the selection threshold).

10 Feature passing (Bresnan, 1982; Gadzar et al., 1985; Pollard and Sag, 1994) is a mechanism that supports the passing of information about a moved constituent across the parse tree. It is the process that implements agreement, control, long-distance movement, subcategorization, selection and other grammatical constraints in Unification Grammars (Francez and Wintner, 2012) and it does essentially the same work in SOSP. A special process of *erroneous feature percolation* (based on Unification Grammar feature passing) has been invoked under the Marking and Morphing Theory of language production (Eberhard et al., 2005) to handle agreement attraction phenomenon in complex noun phrases like *the key to the cabinets*. Although erroneous feature percolation seems akin to Unification Grammar feature passing, it is different in critical ways. Unlike erroneous feature percolation, feature passing is not limited to the subject node but happens across the syntactic tree; it is not limited to number features but encodes all features that must be coordinated across a tree; it spreads both down and up, not only up. Also, most importantly, while erroneous feature percolation violates grammatical constraints, and is thus not independently motivated by linguistic theory, Unification Grammar feature passing is broadly effective in capturing linguistic facts (see references above).

11 In most trials, for sentences in the form of our stimuli, the actual subject wins the competition to be the subject because the subject slot is made available by the verb first, and the subject noun phrase is more activated at that moment than the object noun phrase, because the subject has just been perceived (similarly to what happens in ACT-R as we described above).

12 This idea is similar in spirit to Ferreira’s hypothesis that the parser may first engage in a shallow processing of the sentence, possibly because this process is more efficient and less resource-consuming, and would only stabilize on the structure once a question is asked and requires that a decision be taken (e.g., Ferreira, 2003).

13 One may wonder why the effect of verb marking (the stronger effect of match found for present than past verbs) appeared on-line but not off-line. This observation receives a natural explanation under SOSP, because what matters off-line is which features have gotten onto the verb, and not whether they have originally been encoded on the verb due to morphology or due to being transferred during on-line processing.
